# Vaccination Strategies against Highly Pathogenic Arenaviruses: The Next Steps toward Clinical Trials

**DOI:** 10.1371/journal.ppat.1003212

**Published:** 2013-04-11

**Authors:** Stephan Ölschläger, Lukas Flatz

**Affiliations:** Department of Dermatology, University Hospital of Lausanne CHUV, Lausanne, Switzerland; University of Alberta, Canada

## Abstract

Vaccination is one of the most valuable weapons against infectious diseases and has led to a significant reduction in mortality and morbidity. However, for most viral hemorrhagic fevers caused by arenaviruses, no prophylactic vaccine is available. This is particularly problematic as these diseases are notoriously difficult to diagnose and treat. Lassa fever is globally the most important of the fevers caused by arenaviruses, potentially affecting millions of people living in endemic areas, particularly in Nigeria. Annually, an estimated 300,000 humans are infected and several thousands succumb to the disease. The successful development of the vaccine “Candid#1” against Junin virus, the causative agent of Argentine hemorrhagic fever, proved that an effective arenavirus vaccine can be developed. Although several promising studies toward the development of a Lassa fever vaccine have been published, no vaccine candidate has been tested in human volunteers or patients. This review summarizes the immunology and other aspects of existing experimental arenavirus vaccine studies, discusses the reasons for the lack of a vaccine, and proposes a plan for overcoming the final hurdles toward clinical trials.

## Literature Search

The literature search was based on PubMed, Embase, and Web of Science. The initial search term used was “Lassa OR Junin OR Machupo OR Guanarito OR Sabia AND (vaccine OR vaccination).”

Titles and abstracts were screened to exclude irrelevant publications.

## Introduction

**Figure 1:**
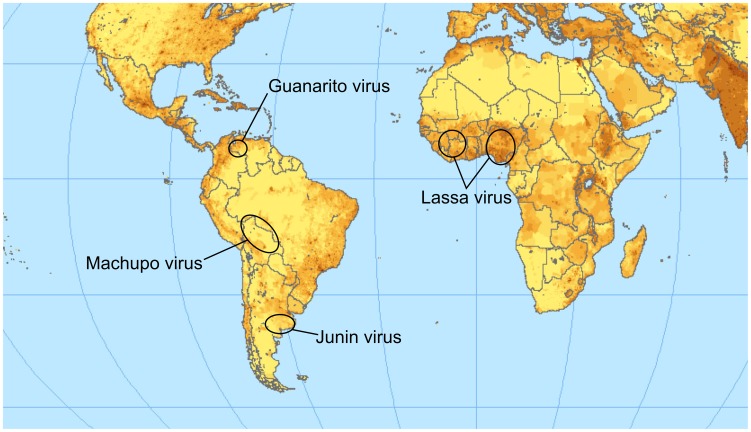
Endemic regions for the pathogenic arenaviruses mentioned in the text. The color intensity indicates the population density. Lassa virus is clearly endemic in the most populated region of Africa; a vaccine is therefore of high relevance for public health. The map is available under a Creative Commons license (http://www.flickr.com/photos/54545503N04/5485517485/sizes/o/in/photostream/).

The family *Arenaviridae* contains four important species that cause severe hemorrhagic zoonoses in humans. Together, they have an important impact on public health in endemic regions (Figure 1). Lassa virus (LASV) is endemic to Africa. The other three species (Machupo, Junin, and Guanarito viruses [MACV, JUNV, and GTOV, respectively]) are from South America [1]. The prototypic arenavirus is lymphocytic choriomeningitis virus (LCMV), which can also cause disease in humans, especially in immunocompromised patients [2].

Arenaviruses carry two RNA genome segments (small, S, and large, L), which encode two genes each [3]. The S-segment encodes the glycoprotein precursor (GPC) and, in ambisense, the nucleoprotein (NP). Similarly, the L-segment encodes the matrix protein Z and, in negative sense, the multifunctional protein L [4].

Natural reservoirs include different species of rodents, depending on the arenavirus [5]. The exact mode of transmission to humans is unknown but probably occurs through direct contact with the infected host or food contaminated with excrement. Direct human-to-human transmission is possible and regularly occurs in clinical settings in endemic areas [6].

Little is known about the pathogenesis of the diseases caused by arenaviruses. A putative explanation for the severe symptoms is an immunopathology caused by an imbalanced host–pathogen interaction with a perpetuated excessive reaction of host immune cells combined with delayed viral clearance [7]. Furthermore, early immune evasion may participate in the disease through delayed virus clearance [8].

Treatment options for the patients are limited. In addition to intensive care, the broad-band antiviral drug ribavirin has proven to be effective if administered early in the course of the disease (before day 6) [9]. The caveat is the need for early diagnosis, and this is a genuine problem, since infections with arenaviruses are initially often mistaken for malaria, typhoid fever, or other common tropical diseases due to the nonspecific nature of the symptoms [10], [11].

The only currently available vaccine is Candid #1. This attenuated JUNV strain was generated through multiple passaging and provided good protection in clinical trials against argentine hemorrhagic fever (AHF) with an excellent safety profile [12]. The historical development and biological properties of this vaccine were recently reviewed in a concise overview [13].

Although there has been much effort to develop vaccines against LASV, none have been effective enough to warrant clinical trials. In this review, we summarize the work that has been done toward the development of vaccines against hemorrhagic fever caused by arenaviruses and discuss the current obstacles toward a licensed vaccine.

## Immunological Basis

### Neutralizing Antibodies

The role of neutralizing antibodies (nAbs) in the control of arenavirus infections is controversial, but has been studied for LASV and JUNV in both human patients and animal models. The use of convalescent plasma has also been studied. Monkeys and guinea pigs are protected against Lassa fever by treatment with plasma from convalescent animals containing high titers of nAb [14], [15]. To be protective, however, the plasma had to be administered directly after infection. Treatment after the onset of symptoms was not beneficial. The time point for successful application could be delayed by using a combination of plasma and ribavirin in experimental settings [16], but the treatment of Lassa fever patients with plasma of survivors did not confer protection [9]. The treatment of these patients with convalescent plasma was initiated within 24 h after admission to hospital. The patients were subdivided into two groups. One group was treated before day 6 after onset of symptoms and the second group after 6 days of disease. No beneficial effects were observed in either group.

In a placebo-controlled treatment study of JUNV, however, convalescent plasma of human AHF survivors benefited AHF patients with acute disease [17]. Similarly to the above-mentioned LASV study, treatment was initiated only after onset of symptoms. In contrast to the case of LASV, however, mortality caused by JUNV was reduced if treatment began within 8 days of onset of symptoms. The difference in efficacy for LASV versus JUNV infections therefore seems unrelated to the time at which treatment was initiated. The divergence in study outcomes could be explained by the presence (JUNV) or absence (LASV) of nAbs in convalescent serum. However, the serum was not assayed for neutralizing antibodies.

The role of nAbs during vaccination and natural infection has also been investigated.

Heterologous vaccination with an apathogenic, related virus has been tested for two pairs: LASV/Mopeia virus (MOPV) and JUNV/Tacaribe virus (TACV). Cross-protection was demonstrated for LASV/MOPV without the appearance of nAbs [18].

In the case of JUNV/TACV, two groups [19], [20] showed that TACV infection leads to heterologous protection of guinea pigs against JUNV challenge. However, the first group could not detect substantial amounts of heterologously cross-reacting and neutralizing antibodies after a single vaccination. In a later study, JUNV cross-neutralizing antibodies were detected 65 days after immunization with TACV in guinea pigs [21].

Experiments in marmosets confirmed cross-protection between TACV and JUNV [22]. After vaccination and before JUNV challenge, no cross-reactive nAbs were detectable in TACV-immunized nonhuman primates. Homologous nAbs could only be measured after infection with JUNV. The authors concluded that cellular immunity may play an important role in protection. Furthermore, in another study, guinea pigs were not protected against the lethal XJ strain after vaccination with formalin-treated JUNV, despite the detection of nAbs before challenge [23]. Finally, Lopez and colleagues [24] measured nAbs after immunization with recombinant vaccinia virus expressing either TACV or JUNV GP, but the role of the nAbs in protection against a lethal JUNV challenge in guinea pigs was unclear. In summary, nAbs can prevent infection and play a role in arenavirus clearance, at least for JUNV infections. Nevertheless, the absence of nAbs in animals that were protected does suggest that cellular immunity is important for protection against both JUNV and LASV infections.

### T-Cell Epitopes

Knowledge of protective T-cell epitopes is essential for the development of vaccines based on cellular immunity.

Ter Meulen and colleagues screened for T-cell clones in Lassa fever survivors using recombinant antigen [25] and identified five Human Leukocyte Antigen DR-1 (HLA-DR)–restricted epitopes in the nucleoprotein of LASV (aa 176–188, 190–202, 288–300, 379–391, and 498–510) that could induce the proliferation of peripheral blood mononuclear cells (PBMCs) from donors in vitro. Proliferating cells were CD4+. The authors also checked for cross-reactivity with homologous epitopes from MOPV and a Nigerian LASV strain. They found partial cross-reaction of T-cells from two of three tested donors. In another study, based on computational epitope predictions, MHC I (HLA-A02) LASV NP and GPC immunogenic peptides were identified [26]. Some of these predicted epitopes could be used to elicit a CD8+ response in HLA-A02 transgenic mice. Botten and colleagues designed a study system using HLA-A02 transgenic mice and challenged peptide-immunized animals with rVaccinia virus expressing LASV GP or NP. This elegant method allowed in vivo screening outside of a BSL-4 setting not only for immunogenic but also for protective T-cell epitopes. However, it remains a limitation that the challenge was performed with a virus from another family expressing LASV antigens rather than LASV itself [27]. The authors identified highly protective epitopes that were conserved among different LASV strains. The crucial question, however, is whether there are epitopes in humans allowing reliable cross-protection between virus strains or even species. The question of cross-reactivity is important, as the sequence variability of arenaviruses in general, and of LASV in particular, is very high. Insufficient cross-protection could in the worst case lead to a reduced immune response due to original antigenic sin [28]. One epitope in LASV GPC (403–417) was found that elicited cross-protective CD4+ cells against LCMV in C3H/HeJ (H-2^k^) mice [29]. Also, Oldstone et al. (2001) found that the H-2^d^ restricted epitope NP 118–126 of LCMV induces cross-protection to other Old World arenavirus homologues but not to the New World homologue [30].

A human T-cell epitope identified at position 289–301 in LASV GPC is highly conserved within Old and New World arenaviruses. CD4+ T-cells from a Lassa fever reconvalescent donor reacted to this epitope when the sequences were changed to the homologue sequences of other arenaviruses [31].

In another study, HLA-A02 and HLA-A03 restricted, protective epitopes were identified for different arenaviral pathogens [32]. The authors proved the feasibility of inducing a multivalent T-cell response by using a cocktail of epitopes from different arenavirus species to create broad protection. They also addressed the question of the distribution of MHC haplotypes in different ethnic populations and calculated the population coverage. This was done based on allele frequency and binding affinity data. Later, the study was extended to focus on single epitopes rather than mixing species-specific epitopes that protect across species borders [33].

In Tables 1 and 2, we summarize the known epitopes for CD8 and CD4, respectively. None of the mentioned studies demonstrated an increased severity of pathology, as would be expected in the case of incomplete protection. However, the current data do not allow a clear prediction of whether a T-cell–based vaccine will increase the risk of immunopathology in clinical trials. In the case of LCMV Armstrong Cl13, which establishes a persistent infection in mice, the number of specific T-cells at the time of infection can determine different disease outcomes. The presence of a low amount of specific T-cells resulted in persistence, whereas a medium amount of specific T-cells led to immunopathology. Complete virus clearance was seen only in the presence of a high number of T-cells [34]. Based on this dataset, a mathematical model for protection, T-cell pathology, and viral persistence was created [35].

**Table 1:** Human HLA class I restricted epitopes against pathogenic arenaviruses.

Epitope	Peptide Sequence	MHC I Restriction	Reference(s)
GTOV			
GPC_427–435_	GTTSLFLHL	HLA-A2	[33]
L_1977–1985_	ATVKNVVLR	HLA-A*1101	[32]
JUNV			
GPC_18–26_	ALNIALVAV	HLA-A*0201	[32]
LASV			
GPC_42–50_	GLVGLVTFL	HLA-A*0201	[27]
GPC_60–68_	SLYKGVYEL	HLA-A*0201	[26], [27]
GPC_111–120_	SIINHKFCNL	HLA-A*0201	[32]
GPC_441–449_	YLISIFLHL	HLA-A*0201	[26], [27]
LCMV			
GPC_10–18_	ALPHIIDEV	HLA-A*0201	[76], [77]
GPC_11–19_	LPHIIDEVI	B*4402	[77]
GPC_38–46_	FATCGIFAL	B*0702	[77]
GPC_46–55_	LVSFLLLAGR	HLA-A*1101	[32]
GPC_112–120_	FTNDSIISH	HLA-A*1101	[32]
GPC_447–455_	YLVSIFLHL	HLA-A*0201, B*1501	[76], [77]
NP_45–53_	SEVSNVQRI	B*4402	[77]
NP_69–77_	SLNQTVHSL	HLA-A*0201	[76], [77]
NP_246–254_	AAVKAGAAL	B*0702	[77]
NP_414–422_	KQFKQDSKY	B*1501	[77]
Z_24–33_	TTYLGPLSCK	HLA-A*1101	[32]
Z_49–58_	YLCRHCLNLL	HLA-A*0201	[32], [76], [77]
MACV			
GPC_18–26_	ALNIALVAV	HLA-A*0201	[32]
GPC_444–452_			[33]
NP_19–27_	GLSQFTHTV	HLA-A*0201	[32]
NP_82–90_	SIQKNTIFK	HLA-A*1101	[32]
NP_432–440_	AMPGVLSYV	HLA-A*0201	[32]
Z_27–36_	RTAPPSLYGR	HLA-A*1101	[32]

Italics, predicted restriction only. GTOV, Guanarito virus; JUNV, Junin virus; LASV, Lassa virus; LCMV, lymphocytic choriomeningitis virus; MACV, Machupo virus.

**Table 2:** Human HLA class II restricted epitopes against pathogenic arenaviruses.

Epitope	Peptide Sequence	MHC II Restriction	Reference(s)
LASV			
GPC_236–250_	PSPIGYLGLLSQRTR	HLA-DRB1*0101	[78]
GPC_241–255_	YLGLLSQRTRDIYIS	HLA-DRB1*0101	[78]
GPC_282–294_	RWMLIEAELKCFG	HLA-DRB	[31]
GPC_289–301_	ELKCFGNTAVAKC	HLA-DRB	[31]
GPC_394–406_	LNETHFSDDIEQQ	HLA-DRB	[31]
GPC_476–490_	SCGLYKQPGVPVRWK	HLA-DRB1*0101	[78]
NP_176–188_	FGTMPSLTLACLT	HLA-DRB	[79]
NP_190–202_	QGQVDLNDAVQAL	HLA-DRB	[79]
NP_288–300_	ALGMFISDTPGER	HLA-DRB	[79]
NP_379–391_	QLDPNAKTWMDIE	HLA-DRB	[79]
NP_498–510_	VWDQYKDLCHMHT	HLA-DRB	[79]
GTOV			
GPC_131–145_	KGSPEFDWILGWTIK	HLA-DRB1*0101	[78]
L_181–195_	DQEYHRLIHSLSKTS	HLA-DRB1*0101	[78]
L_391–405_	RVLDILVARRLLLKK	HLA-DRB1*0101	[78]
L_1826–1840_	IQLVFSSMINPLVIT	HLA-DRB1*0101	[78]
NP_166–180_	KLNNQFGSMPALTIA	HLA-DRB1*0101	[78]
NP_191–205_	NNVVQALTSLGLLYT	HLA-DRB1*0101	[78]
NP_236–250_	ISGYNFSLSAAVKAG	HLA-DRB1*0101	[78]
NP_541–555_	IPIQLLPNTLVFQAK	HLA-DRB1*0101	[78]
JUNV			
GPC_46–60_	FFVFLALAGRSCTEE	HLA-DRB1*0101	[78]
L_381–395_	VGQMLMLVNDRLLDI	HLA-DRB1*0101	[78]
L_391–405_	RLLDILEAIKLIRKK	HLA-DRB1*0101	[78]
L_411–425_	KWVQMCSRTLKNSHQ	HLA-DRB1*0101	[78]
L_1491–1505_	MFIRNCARKVFNDIK	HLA-DRB1*0101	[78]
L_1711–1725_	NKNFFWAVKPKAVRQ	HLA-DRB1*0101	[78]
LCMV			
GPC_66–80_	DIYKGVYQFKSVEFD	*0701	[77]
GPC_71–85_	VYQFKSVEFDMSHLN	*0701	[77]
GPC_341–355_	HLFKTTVNSLISDQL	*0701	[77]
GPC_421–435_	LRKDYIKRQGSTPLA	HLA-DRB1*0101	[78]
L_256–270_	RNFQKVNPEGLIKEF	HLA-DRB1*0101	[78]
L_946–960_	HLRKVILSEISFHLV	HLA-DRB1*0101	[78]
NP_6–20_	EVKSFQWTQALRREL	HLA-DRB1*0101	[78]
NP_86–100_	KNVLKVGRLSAEELM	*1101	[77]
NP_106–120_	LEKLKAKIMRSERPQ	*0801	[77]
NP_236–250_	NISGYNFSLGAAVKA	*0701	[77]
NP_261–275_	LESILIKPSNSEDLL	*0701	[77]
NP_281–295_	AKRKLNMFVSDQVGD	*0701	[77]
NP_311–325_	EGWPYIACRTSIVGR	*0701	[77]
NP_356–370_	VGLSYSQTMLLKDLM	*0701	[77]
NP_411–425_	VDQKQFKQDSKYSHG	*0701	[77]
NP_521–535_	MDCIIFESASKARLP	HLA-DRB1*0101, *0801	[77], [78]
MACV			
GPC_96–110_	NSFYYMKGGVNTFLI	HLA-DRB1*0101	[78]
GPC_251–265_	SKTHLNFERSLKAFF	HLA-DRB1*0101	[78]
GPC_446–460_	ASLFLHLVGIPTHRH	HLA-DRB1*0101	[78]
L_391–405_	DRVLDILEAVKLIRK	HLA-DRB1*0101	[78]
L_636–650_	RYFLMAFANQIHHID	HLA-DRB1*0101	[78]
L_866–880_	DYLILKNLTGLVSAG	HLA-DRB1*0101	[78]
L_1491–1505_	TSFIRNCARKVFNDI	HLA-DRB1*0101	[78]
L_1711–1725_	NNQNFFWAVKPKVVR	HLA-DRB1*0101	[78]
NP_191–205_	NSVVQALTSLGLLYT	HLA-DRB1*0101	[78]
Z_21–35_	PSAEFRRTAPPSLYG	HLA-DRB1*0101	[78]

Italics, predicted restriction only. GTOV, Guanarito virus; JUNV, Junin virus; LASV, Lassa virus; LCMV, lymphocytic choriomeningitis virus; MACV, Machupo virus.

## Experimental Vaccines for JUNV and LASV

The most relevant vaccine trials are summarized in Table 3.

**Table 3:** Summary of selected animal JUNV and LASV vaccine trials.

Vaccine	Antigen	Challenge Virus	Protection	Animal Models Tested	Safety	Reference(s)
Candid#1	All homologous	JUNV	Full	Already in use in humans	Safe in humans	Reviewed in [13]
ML-29	NP/GPC (LASV); L/Z (MOPV)	LASV	Full	Guinea pig, nonhuman primates, mouse	Safe in primates	[43], [44]
MOPV	All (MOPV)	LASV	Full	Nonhuman primates	Safe in primates	[18], [40], [41]
TACV	All (TACV)	JUNV	Full	Nonhuman primates, guinea pig	Safe in primates	[20], [21], [22]
rVaccinia virus	NP (LASV)	LASV	Full	Guinea pig	Vector is safe^1^	[57]
rVaccinia virus	NP (LASV)	LASV	None	Nonhuman primates	Vector is safe^1^	[59]
rVaccinia virus	GPC (LASV)	LASV	Partial (86%)	Nonhuman primates	Vector is safe^1^	[59]
rVaccinia virus	GPC (LASV)	JUNV	Partial (72%)	Guinea pig	Vector is safe^1^	[24]
rYellow fever virus 17d	GP1+GP2 (LASV)	LASV	Partial (83%)	Guinea pig	Vector is safe^2^	[64]
rVesicular stomatitis virus	GPC (LASV)	LASV	Full, transient viremia	Nonhuman primates, single human application	Safe in primates	[60], [61]
rVenezuelan equine encephalitis virus	GPC+NP (LASV)	LASV	Full, very low viremia	Guinea pig	Safe in primates	[66]
r*Salmonella typhimurium*	NP (LASV)	LCMV^3^	Partial (33%)	Mouse	Vector is safe^4^	[50]
r*Salmonella typhimurium*	NP (LCMV)	LCMV^3^	Full	Mouse	Vector is safe^4^	[50]
LASV VLPs	All (LASV)	LASV	Not tested	Mouse	nd (not infectious)	[46]
ã-irradiated virus	All (LASV)	LASV	None	Nonhuman primates	Safe in primates	[47]
Formalin-treated virus	All (JUNV)	JUNV	None	Guinea pig	Safe in Guinea pigs	[23]
DNA vaccine	NP (LCMV)	LCMV^3^	Titer reduction	Mouse	Nd (not infectious)	[69]

1 Vaccinia virus has been used for human application.

2 Yellow fever virus 17D has been used for human application.

3 Virus used for challenge. Aim of the study was to explore the use of this method for vaccination against LASV infection.

4 Safety tests for attenuated *Salmonella typhimurium* expressing hepatitis B virus antigens have been done in human volunteers. JUNV, Junin virus; LASV, Lassa virus; LCMV, lymphocytic choriomeningitis virus; MOPV, Mopeia virus; TACV, Tacaribe virus.

### Junin Virus Candid #1 (C#1)

A successful example of a live vaccine for an arenavirus is JUNV Candid#1 (C#1), which confers reliable and safe protection against severe AHF. It is licensed in Argentina for the vaccination of people living in high-risk areas. A recent review summarizes the historical development and biological properties of the vaccine [13]. Using recombinant viruses, a single amino acid in the transmembrane domain of the GPC was found to be responsible for the attenuated phenotype of C#1. A change from phenylalanine to isoleucine on position 427 of GPC (F427I) attenuated the phenotype in mice and led to increased survival upon challenge [36]. Until now, no mechanistic explanation for the attenuation has been found.

### Apathogenic Arenaviruses as Live Vaccines

The use of live-attenuated strains or related apathogenic viruses for vaccination has a long history, starting with vaccinia virus for the prevention of smallpox or the yellow fever virus 17D strain [37], [38]. Both elicit a powerful response of neutralizing antibodies against the agent but also effectively target cellular adaptive immunity [39]. The greatest advantage of live vaccines is the complete activation of several immune pathways, which is analogous to what happens during natural infection. Initial animal experiments suggested that the use of genetically close apathogenic arenavirus species for vaccination against AHF and Lassa fever could be effective. Beginning in the 1970s, the first successful LASV vaccination experiments were done with MOPV in rhesus monkeys [18], [40]. The animals showed no signs of disease after MOPV infection and survived an otherwise fatal infection with LASV. Previously, similar experiments had been conducted with TACV and JUNV in guinea pigs [20]. Unfortunately, very little is known about the infection of humans with MOPV and TACV. It is unclear whether the infection of humans with these viruses is truly apathogenic, as some of the monkeys infected with MOPV showed pathological alterations of the liver and kidney [41]. It will be important to prove the safety of apathogenic arenaviruses in humans before vaccine candidates can be tested for efficacy.

### Reassortment of LASV and MOPV

An interesting live-attenuated vaccine candidate is the chimeric virus ML-29. Lukashevich generated a recombinant virus carrying the LASV S-segment and the MOPV L-segment by co-infection of Vero cells with both virus species [42]. The use of a plaque-purified clone (ML29) as a vaccine against LASV showed promising results [43]. While MOPV vaccination was also protective, only ML29 vaccinated animals did not show a transient elevation of liver enzymes in plasma after LASV challenge. Immunity was conferred through cellular responses while the humoral response was negligible (nAb titer<1∶20). In this and a following study, the recombinant ML29 proved safe in nonhuman primates [44]. The attenuated phenotype is attributed to the presence of the L-segment of MOPV in the recombinant, as no other differences in sequence were detected with respect to corresponding wild-type strains [45].

### Inactivated or Dead Vaccines

Virus-like particles containing GP1, GP2, NP, and Z [46] were produced after transient transfection of expression plasmids into HEK-293T cells. Binding antibodies as determined by ELISA were induced. The protectiveness of the vaccine, and whether or not these antibodies were neutralizing, was not investigated. Further functional experiments will be necessary to predict whether or not this approach will be successful.

Gamma-irradiated LASV did not protect rhesus macaques against challenge, despite an increase in anti-LASV antibodies. This was attributed to the lack of adequate cellular immunity [47]. Guinea pigs vaccinated with formalin-inactivated Junin virus produced neutralizing antibodies but were not protected upon challenge [23]. These studies indicate that a certain level of antigen expression is mandatory to elicit immunity and that cellular immunity may be responsible for clearance of the virus.

In contrast to these findings, Amanna et al. recently reported a LCMV vaccine based on purified LCMV particles inactivated by 3% H_2_O_2_. The vaccine induced T-cell responses when high concentrations (50 µg) of inactivated virus were used in mice [48].

### Mucosal Vaccination

An attractive way to deliver antigen for vaccination is oral uptake. In many instances, the mucosa is the first part of the host to come into contact with a pathogen. Oral application is furthermore minimally invasive and vaccination campaigns can be performed with less effort. *S. typhimurium* and vaccinia virus were genetically modified to express LASV NP and LCMV NP. Mice inoculated with a recombinant vector showed LASV NP–specific IgA and specifically reactive splenocytes [49]. Challenge experiments involving intracerebral LCMV infection after intragastric immunization with vectors expressing LASV antigen protected only approximately one third of the LCMV-challenged animals [50]. The authors concluded that this was due to the use of a heterologous antigen (LASV antigen against LCMV challenge) because intragastric infection of mice with vector expressing LCMV NP before intracerebral challenge led to 100% survival of the animals in the same setting. The idea of intragastric immunization is particularly attractive when immunization campaigns in wild animals are considered (*Mastomys natalensis*); vaccination could be accomplished in a manner similar to that of rabies vaccination in foxes [51].

### Recombinant Viruses Expressing Arenavirus Proteins

Recombinant virus vectors have been in use since the early 1980s [52], [53]. Their use as vaccine vectors for the expression of foreign antigens is a valuable tool and has several advantages compared with other vaccine platforms.

The biggest advantage is the use of genetically defined material. The antigen can be optimized to elicit the desired immune response. Safety aspects, such as the production of replication-incompetent viruses, are more easily addressed [54]. There is no need to rely on the random mutations required for the generation of attenuated WT strains.

The different virus platforms allow the selection of a vector that has a similar tropism to that of the target virus (e.g., recombinant vaccinia virus for intradermal vaccination or recombinant influenza virus for targeting the respiratory tract mucosa [55], [56]).

For arenavirus, different recombinant virus platforms have been tested. Vaccinia virus (VV) vectors have been modified to express LASV NP [57] and GPC [58]. Both antigens protected guinea pigs against a lethal challenge with LASV. Similar experiments have been performed for JUNV [24], but the guinea pigs vaccinated with recombinant VV expressing JUNV-GP were not fully protected (72% survival) and protection after vaccinations with TACV antigens (GPC and NP) had even poorer outcomes (50% and 0% protection, respectively). Interestingly, neutralizing antibodies again seemed to play no obvious role in the protected animals. In a series of experiments in nonhuman primates, the roles of different LASV antigens were tested. Vaccinia virus vectors expressing only the N-terminal (GP1) or the C-terminal (GP2) part of GPC or the NP were produced. Animals that received a vector expressing only GP1 or GP2 succumbed to disease upon challenge. Similarly, vaccination with the NP-only vector did not confer significant protection. Only when the whole glycoprotein or both parts were used for immunization were animals significantly protected against challenge [59]. The experiments were performed using rhesus and cynomolgus macaques with comparable results. The authors concluded that similar observations could be expected in humans.

In 2004, the construction of a recombinant vesicular stomatitis virus (rVSV) carrying LASV glycoprotein was described [60]. The growth of the virus with the foreign GP was attenuated in mice compared with the wild-type vector. In challenge experiments in nonhuman primates, the vector protected animals against lethal LASV infection. However, the protection was not sterile and LASV viremia could be measured on day 7 postinfection. The viremia at that time point was comparable to that of control animals that received an irrelevant immunization. Nevertheless, vaccinated animals showed no signs of disease and clearance of the virus shortly afterwards [61]. The rVSV vaccine backbone has already been used in one human case of accidental Ebola exposure. The patient showed a transient elevation of body temperature, and vaccine vector RNA was detected by PCR in the blood. No other severe side effects were observed. Together with data available from monkey experiments, the safety of the vector seems to be good [62].

The attenuated yellow fever strain 17D (YFV 17D) is one of the oldest and most successful virus vaccines ever used. Use of recombinant YFV 17D is therefore promising because its safety and potential to elicit immunity are known. The addition of foreign genes to the YFV genome further attenuates the replication capacity of the virus. Some recombinant YFV 17D-based vaccines are already in clinical trials. A general review of YFV 17D vectors has been published recently [63]. Two studies described the use of YFV 17D–expressing LASV GP or GP1 and GP2 for vaccination of guinea pigs [64], [65]. While the overall survival was 80% all animals showed viremia and developed disease. Furthermore, the use of a vaccine backbone with a known safety profile would significantly facilitate its use in a clinical trial.

Another vaccine vector that proved effective in guinea pigs against LASV challenge is a Venezuelan equine encephalitis virus (rVEE) replicon particle expressing GP or NP [66]. Animals were fully protected against LASV challenge after prime/boost/boost immunization with this vector. A recombinant LCMV expressing LASV antigens has recently been described [67]. The use of modified apathogenic arenaviruses could be another option for the development of vaccines against pathogenic relatives of arenaviruses.

### DNA Vaccine

Plasmid DNA can be used to express and deliver target genes into the host to induce immunity. Antigen-presenting cells and other body cells take up plasmid DNA and the ensuing protein synthesis from the plasmid DNA leads to MHC I and II presentation of peptides encoded by the plasmid DNA [68]. Whitton et al. have extended their extensive work on LCMV to the highly pathogenic LASV [69]. After injecting plasmid DNA encoding LASV or LCMV NP into mice, they measured the immune response and protective potential upon challenge with LCMV or Pichinde virus (PICV). LCMV virus titers were lower in vaccinated than in naïve animals after challenge, irrespective of which antigen was used. This indicates cross-species protection. Even PICV titers were lower after LCMV and LASV NP immunization.

Although DNA vaccines have an excellent safety profile in humans, their rather weak immunogenicity may require prime-boost vaccination with other vectors.

## Conclusion and Discussion

The development of vaccines against arenaviruses started shortly after the identification of JUNV as the causative agent of AHF in the 1950s. These efforts culminated in the introduction of the licensed Candid#1 vaccine in the 1990s, which led to a significant decrease in mortality and morbidity to AHF [12].

We will focus on LASV in the Discussion because it is by far the most important pathogenic arenavirus clinically and has an important impact on public health. Approximately 100,000 to 300,000 cases including several thousand fatal outcomes are estimated to occur annually in West Africa [70]. Much effort has been put into the development and preclinical testing of LASV vaccines, as revealed by the number of publications on this topic. Many different vaccine vectors and platforms have been evaluated for use as a LASV vaccine (Table 3). We therefore raise the question of why not a single vaccine system has entered a clinical trial. We discuss the hurdles clinicians face in treating these diseases and how these hurdles could be surmounted.

Firstly, LASV is a neglected tropical disease. Affected countries are Nigeria, Ivory Coast, Mali, Guinea, Sierra Leone, and Liberia. The specific socioeconomic problems related to LASV vaccination are probably the biggest hurdles to be crossed. People who are at the highest risk of infection are also the poorest in the above-mentioned endemic countries. Poor hygiene and sanitation in these countries increases the probability of LASV exposure. Furthermore, many people living in these countries are unable to pay for vaccination. Prime-boost vaccination campaigns are difficult to organize due to the poor infrastructure. The low commercial value of a LASV vaccine makes the development of such vaccines an unattractive prospect for pharmaceutical companies. Instead, international nongovernmental organizations (e.g., WHO and the World Bank) and foundations must provide the necessary financial support. Governments of the endemic countries should also contribute by logistically supporting clinical investigations in LASV patients and helping to organize phase II clinical trials for vaccine studies after phase I safety trials have been conducted in Western countries. As Nigeria is the biggest local economic power and among the top 10 oil-producing countries worldwide, it could take a substantial lead in these actions (World Bank, GDP ranking 2011 [71]; OPEC market indicators, December 2012 [72]).

Secondly, the mechanisms underlying protection are still unclear. It seems to be the consensus that nAbs play no major role in the clearance of LASV. The specific role of T-cells in LASV infections, however, remains speculative [73]. In humans, only a few studies investigating the role of T-cells in Lassa fever outcome exist. The efficacy of cross-protection between different LASV strains by a single vaccine or a mixture remains to be determined.

Even for JUNV, no complete picture of the mechanisms that underlie protection through vaccination is available. nAbs are often seen as a correlate of protection against disease. The role of T-cells has not been studied in much detail and the role of nAbs in JUNV infections may have been overestimated. Therefore, we hypothesize that the contribution of T-cells to virus clearance is stronger than currently assumed. The many studies showing protection through vaccination without detectable nAb titers before infection support a stronger role for T-cells than previously thought. In our opinion, there is a need for the development of simple “bed-side” T-cell assays to study the role of T-cells. One could imagine assays similar to the interferon-γ release assay for tuberculosis [74]. With simple T-cell assays like this, T-cell presence in vaccinated persons and patients could be determined. It would be interesting to see how this would correlate with protection and recovery, and compare with the protection and recovery provided by the antibody response.

Thirdly, it is still unknown what role T-cells play in arenavirus pathogenesis. T-cell–mediated pathology is a mechanism that could possibly play a role in the development of severe arenaviral disease. We recently showed that T-cells play a major role in causing immunopathology in a mouse model for LF using mice transgenic for HLA-A02 [7]. Until now, it has been unclear whether vaccine-related T-cell pathology can occur following imperfect vaccination. If it does, it could have adverse consequences for clinical trials of these vaccines in humans.

Fourthly, the safety aspects of vaccination campaigns have to be considered. Besides the more general safety concerns linked to vaccination, the issue of HIV co-infection is of high importance. LASV-endemic regions are also regions with a high HIV prevalence rate. There are no data available on Lassa fever in relation to HIV. The safety of vaccination with live-attenuated vaccines in immunocompromised individuals is unclear. A recent meta-analysis on the use of the yellow fever vaccine YF17D in immunocompromised patients [75] did not show a high risk of severe adverse effects in HIV-infected individuals. Nevertheless, the test groups were quite small and more information will be needed to draw firm conclusions about safety.

We propose the following steps to move closer to clinical trials of LASV vaccines: (i) Studies of the role of T-cells during pathogenesis (especially for LASV); (ii) identification of the correlate of protection in survivors; and (iii) identification of the most promising vaccine concept (in terms of safety, protective efficacy, and technical feasibility). The scientific community should meet in an international conference on LASV vaccines and discuss the different approaches.

In our opinion, the experimental basis is at a sufficient stage of advancement for clinical trials to proceed. Much work done in different animal species (including nonhuman primates) has demonstrated the safety and efficacy of several vector systems for vaccination against LASV infection.

What will be the most promising vector system for clinical trials? MOPV in humans will probably offer the most effective protection against subsequent LASV infection, but the risks involved in using a nonattenuated BSL-3 agent (BSL-2 in Europe) in clinical trials are currently too high. The same is true for the recombinant MOPV-LASV assortant ML-29. DNA vaccination would be cheap and safe, but it is highly questionable whether a single application would be sufficient for protection. As inactivated virus particles seem to be ineffective, we think that the most promising vaccine will probably be a system based on recombinant virus vectors (e.g., vaccinia virus or yellow fever virus) for which safety data (especially as mentioned above in the context of HIV) are available. Safety aspects can first be assessed in clinical trials outside of West Africa to circumvent political and logistical problems in the endemic countries. We hope to see LASV vaccine candidates moving into clinical trials within the next few years. Clinical approval of a LASV vaccine would not only bring decades of hard work to a successful end but would also justify the resources (time and human resources, animal lives, and money) that have been used.
